# Montreal Cognitive Assessment (MoCA) use in general practice for the early detection of cognitive impairment: a feasibility study

**DOI:** 10.3399/BJGPO.2024.0039

**Published:** 2025-01-29

**Authors:** Cassandre Carton, Matthieu Calafiore, Charles Cauet, Nassir Messaadi, Marc Bayen, David Wyts, Wassil Messaadi, Teddy Richebe, Sabine Bayen

**Affiliations:** 1 District of General Medicine, Faculty of Medicine, University of Lille, Lille, France; 2 University of Lille, CHU Lille, ULR 2694 - MSPU Wattrelos, Lille, France; 3 University of Lille, CHU Lille, ULR 2694 - PSPU Lille, Lille, France; 4 INSERM 1172, University of Lille, Lille, France; 5 University of Lille, CHU Lille, ULR 2694 - MSPU Guesnain, Lille, France

**Keywords:** screening, consultation skills, dementia, cognitive dysfunction, general practice, primary healthcare

## Abstract

**Background:**

GPs can detect cognitive impairment (CI) at a very early stage, allowing early support for people and their caregivers. The early onset of CI is between 50 years and 60 years. Currently, in France, the Mini-Mental State Examination (MMSE) remains the most used screening test, although it has a lower sensitivity and specificity than the Montreal Cognitive Assessment (MoCA) for detecting mild CI, taking an average of 15 minutes to complete.

**Aim:**

To investigate the feasibility of the MoCA during routine consultations in general practice for the early detection of CI and to determine prevalence of CI in a primary care setting.

**Design & setting:**

A quantitative, prospective feasibility study was carried out in real-life working conditions during routine GP consultations in France.

**Method:**

GPs performed MoCA on adults aged ≥50 years, without suspected or confirmed CI.

**Results:**

Sixty-one GPs performed 221 MoCA with a mean duration of 8 minutes and detected mild neurocognitive impairment in 62% of patients.

**Conclusion:**

The MoCA is feasible and easy to perform during routine consultations in general practice by trained and experienced physicians.

## How this fits in

Early screening of mild cognitive impairment (CI) optimises person-centred care. Most GPs in France use the Mini-Mental State Examination (MMSE) to assess CI. However, the Montreal Cognitive Assessment (MoCA) is a reliable screening tool for mild CI that is more sensitive and specific than the MMSE. The study showed that mild CI was prevalent in general practice. Therefore, GPs should be encouraged to use the most appropriate screening tool for CI during routine consultations.

## Introduction

In France, more than three million people were affected by Alzheimer’s disease or a related dementia in 2019, including about 1.2 million patients and 2 million caregivers.^1,2^ It is estimated that 2 million people will be affected by 2050.^1,2^

There is currently no cure for neurodegenerative diseases. In general, the disease onset is slow, allowing people to remain active in society from the outset. Early detection is essential from a medical–social point of view to enable people to maintain independence for as long as possible. GPs have a crucial role to play^3^ by screening for cognitive impairment (CI) to provide early support to the person and caregivers.^4,5^ As the minimum age for the onset of CI ranges from 50 years to 60 years,^6^ it seems appropriate to screen for it at this age.

Several tests are available and the best known are as follows: the Mini-Mental State Examination (MMSE); the General Practitioner assessment of Cognition (GP-COG); and the Montreal Cognitive Assessment (MoCA).

In France, screening for CI in general practice is encouraged and possible for the same person once a year. This dedicated consultation includes not just the cognitive assessment but also the announcement of the results and, if necessary, programming of complementary investigations such as blood analysis or brain magnetic resonance imaging (MRI). The test to assess CI (ALQP006) costs 69.12 Euros (approximately 57.60 GBP), which is completely reimbursed to the patient by the National Health Insurance Fund (NHIF), which covers a limited list of approved tests.^7,8^ To date, the MoCA has not been included in this restrictive list.

In France, most GPs use the MMSE to assess for CI,^5^ among 505 GPs, 76% reported to use it,^9^ since MMSE was recommended by a consensus conference in 2000 for screening for dementia,^10^ needing 10–15 minutes to be administered^11^ by a trained person.^12^

The GP-COG has been validated for screening for CI in general practice. It takes an average of 5 minutes to complete^13^ and is a robust and consistent tool.^14–16^

The MoCA is best suited to screening for mild CI.^12,17^ The MoCA has been compared with the MMSE.^12^ The MoCA is more sensitive and specific than the MMSE for screening for CI in people aged >60 years^12,18^ and for the early detection of mild CI (90% sensitivity compared with 18% for the MMSE). The MoCA is not a substitute for the MMSE, which is suitable for moderate-to-severe CI.^13,17^

No early screening test for CI is recommended in general practice.^12,13^

The primary aim of this study was to investigate the feasibility of using the MoCA during routine consultations in general practice for the early detection of CI. Secondary aims were to identify prevalence of CI in a primary care setting, and to determine any difficulties encountered by GPs and patients in completing the MoCA.

## Method

### Study design

A quantitative, cross-sectional feasibility study was carried out in the Hauts-de-France region between November 2022 and April 2023.

Since GPs prefer to use the MMSE, taking 10–15 minutes to complete, we set the feasibility of the MoCA at <15 minutes. The primary endpoint was the feasibility of the MoCA during a feasibility threshold set at a maximum of 15 minutes. The presence of any difficulties experienced by the GP or patient in performing the MoCA has been reported in an open-ended questionnaire after the test.

### Study population

People aged ≥50 years on the active list of GPs practising in Hauts-de-France participated.

The inclusion criteria were men and women aged ≥50 years who volunteered to take part in the study and who were able to complete the MoCA (having a good command of French) and to follow the instructions of the investigating GP. The non-inclusion criteria were people aged <50 years of age and/or with a history of dementia or CI already diagnosed.

### Recruitment

We invited 301 GPs by email. Their email addresses were obtained through acquaintances and GP networks (GP trainee trainer, university GP teacher). Attached to the email, they received an invitation page explaining the background and interest of this work, a tutorial on how to use and score the MoCA in French (version 8.3), and a patient information page explaining the purpose of the test and how to use it. They were also sent a link to complete the questionnaire online.

### Data collection

An online questionnaire was created on Lime Survey to collect the MoCAs completed by the GPs. The questionnaire consisted of three parts:

1. Sociodemographic data on the GPs: sex, age, practice location (rural, semi-rural, or urban), type of practice (single or group), number of GPs, practice structure, and patient profiles.
2. Data on screening for CI: knowledge and use of the ALQP006 score, reasons for non-use, and opinion on the likely under-screening of CI in general practice.
3. The patient’s sociodemographic data (sex, age, level of education) were then completed before the MoCA was administered. The start and end times of the test were recorded. After the test was completed, the GP reported any difficulties encountered by them and/or the patient. These data were collected using an open-ended questionnaire. For the analysis, we listed each of the difficulties that might have been encountered by the doctor and the patient, and then tallied the responses. The test was scanned into the Lime Survey questionnaire in png, gif, doc, odt, jpg, or pdf format. This last part was repeated if the GP wanted to include more patients (maximum 10 patients).

### Ethics

Patients were informed of the study aim and reminded that this was voluntary, confidential, and anonymous. According to the result of the test, the GP proceeded to complementary investigations, if necessary and accepted by the patient. These investigations could include several steps: a prescription analysis to identify iatrogenic causes for the CI, blood analysis, an audition and vision check, brain MRI followed by neurological visits or not.

### Duration of the study

Data collection began with an email sent on 8 November 2022 and ended on 12 April 2023.

## Results

Of the 301 GPs invited, 61 agreed to participate. A total of 221 MoCAs were carried out.

Table 1 summarises the sociodemographic characteristics of the investigating GPs.

**Table 1.** Sociodemographic characteristics of the investigating GPs

Characteristic	Number of GPs (*n* = 61)	Percentage (%)
**Sex**		
Female	43	70
Male	18	30
**Type of practice**		
Single practice	8	13
In a group	53	87
**Number of GPs in the practice (*n* = 53)**		
2	11	21
3	6	11
4	2	4
5	3	6
7	31	58
**Practice structure**		
Medical practice	43	70
Health centre	2	3
MSP	16	26
**Practice environment**		
Rural	2	3
Semi-rural	48	79
Urban	11	18
**Type of patient (majority)**		
Geriatric	0	0
Paediatrics	1	2
Varied	59	96
Other: gynaecological and paediatric referral	1	2

MSP = Maison de Santé Pluriprofessionelle (Pluriprofessional health care office/praxis/surgery).

All the GPs were self-employed in their private office. We did not recruit any salaried doctors, locums, or GPs with mixed activity. Of all GPs taking part in the study, 70% worked in a GP office, 26% in a multiprofessional primary care (MSP) structure, and 3% in a health centre. For all GPs, the average length of time in practice was 21.7 years (range: 1 year–36 years). Among the 61 GPs, 31 (51%) had already carried out screening tests for CI.

Of the 31 GPs who had performed cognitive screening tests, 19 had performed the MMSE and five the MoCA. The Dubois 5-word test and the clock test had been performed by four and three GPs, respectively. Eleven GPs did not report which tests they had done.

The ALQP006 score was known by 48 GPs (79%). Only half of them used it. Of the 24 GPs who knew about the score but did not use it, two said they did not use it because it was too time-consuming, two others said they had forgotten about it, and 20 said they were not concerned. Under-screening for CI in general practice was reported by all but one of the GPs.

### MoCA analysis

Table 2 shows the patients’ characteristics.

**Table 2.** Characteristics of patients for whom the MoCA was performed

Characteristic	Number of patients (*n* = 221)	Percentage (%)
**Sex**		
Female	147	67
Male	74	33
**Age range of patients, years**		
50–55	29	13
56–60	50	23
61–65	41	19
66–70	40	18
71–75	27	12
76–80	14	6
>80	20	9
**Patients' level of education**		
Study certificate	31	14
French Certificate of General Education	18	8
CAP or BEP	75	34
BAC	28	13
Higher education	52	23
Stopped at 12	2	1
Stopped at 14	3	1
Stopped at 16	5	2
Stopped in 6^ème^	1	1
Did not go to school	2	1
BTS	4	2

6ème = school year, typical age 11–12 years. BAC = Baccalauréat. BEP = Brevet des Etudes Professionnelles (vocational course). BTS = Brevet de Technicien Supérieur (advanced technician's certificate). CAP = Certificat d'Aptitude Professionnelle (certificate of professional competence).

**Figure 1.**
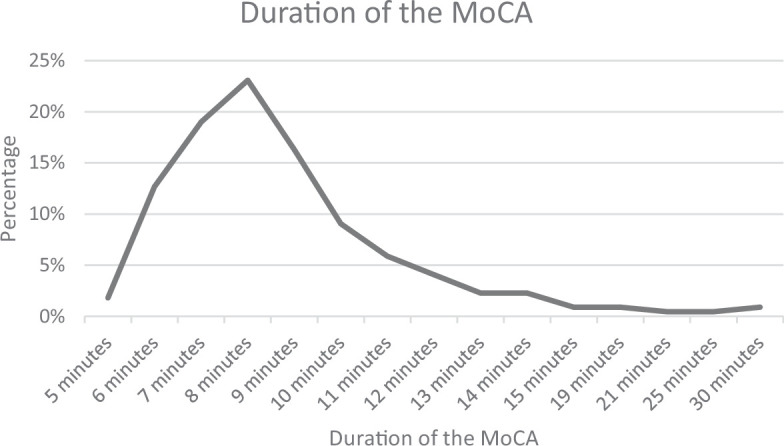
Average time taken to complete the Montreal Cognitive Assessment (MoCA)

The mean age of patients undergoing MoCA was 66 years (range: 50 years–90 years), with 23% in the 56–60 age group. The MoCA took an average of 8 minutes to complete (Figure 1). Eighty-two per cent of the tests were completed in ≤10 minutes and 97% in <15 minutes.

### GPs' feedback

The GPs were asked to answer an open-ended question after the MoCA test about possible difficulties. Through the free commentaries, GPs declared positive experiences of performing the MoCA test especially regarding the easy and quick use of it in daily practice. Three GPs, among those who had already performed cognitive tests in the office, declared they prefer to use the MoCA instead of MMSE since their study participation. Five GPs, among those who had never used cognitive tests before, where happy to discover the MoCA as an easy and reliable tool.

The difficulties encountered by three GPs in carrying out the MoCA, concerned three patients and were related to the patient’s understanding of the instructions and their deafness, which required the instructions to be repeated.

Difficulties encountered by patients (Table 3) could be related to a lack of concentration, sometimes leading to a misunderstanding of the instructions, requiring them to be repeated. This lack of concentration caused them to rush through the exercises, leading to inattention errors. Anxiety about the test, combined with a desire to do well, could make it difficult for the patient. Lack of perseverance or low self-esteem could lead to a loss of motivation to continue with the exercise and thus distort the test result.

**Table 3.** Difficulties encountered by people subject to the MoCA

Type of difficulties encountered by the patient	Number of patients (*n* = 48)	(%)
Lack of perseverance or confidence	8	17
Calculation	3	6
Language	1	2
Indication	1	2
Dyslexia	1	2
Anxiety about taking the test	2	4
Insufficient fluency in French	1	2
Understanding instructions owing to lack of concentration	13	27
Lack of concentration when doing exercises	3	6
Slowness in carrying out exercises, wanting to do things right	3	6
Acute infection	1	2
Hearing problems	6	12
Visual disorders	7	14
Trembling	6	12

Sensory impairments, such as hearing or visual problems or tremor, could make the test difficult to perform, especially if they are not compensated by a hearing aid or optical correction.

Of the 221 MoCAs performed, 137 (62%) identified mild CI (between 18 and 25/30 points in the test), nine (4%) moderate CI (between 10 and 17/30 points in the test), and 75 (34%) normal results (>26/30 points). No patients with severe CI (<10/30 points in the test) were identified.

## Discussion

### Summary

Sixty-one GPs performed a total of 221 MoCA tests among their patients without any prior known CI and 62% of positive tests identified mild CI. This study confirmed MoCA was an easy tool to use during routine consultations in general practice.

The difficulties GPs experienced in performing the MoCA were related to the patient’s deafness. Also, patients reported some difficulties around lack of concentration leading to poor understanding of instructions.

### Strengths and limitations

A more comprehensive approach of data highlighting GPs’ views of the MoCA initiative would have been insightful in terms of explaining why this intervention may or may not have worked well.

The survey period was busy for GPs owing to the winter epidemics and pressures. Therefore, no reminder emails were sent to them, to respect their workload and lack of time.

GPs did not necessarily carry out the MoCA during a specific consultation. People came in for other reasons. Therefore, there was probably a lack of concentration on the part of the patients, who were caught off guard.

In addition, the role of the investigator and bias should be considered. For example, one of the factors for incomprehension was a lack of clarity, which was owing to a lack of time taken by the investigator to give instructions to the participant.

Also, sensory impairments, such as hearing or visual problems, could constitute a functional bias for the patient.

### Comparison with existing literature

Recent studies have focused on the use of cognitive testing in primary care settings to diagnose CI among symptomatic people.^19,20^

We used the MoCA test among apparently asymptomatic people to assess mild CI to promote a very early detection of CI to provide person-centred care to patients and their close family and friends.

Federman *et al* used a similar study design and inclusion criteria to assess the MoCA among a primary care sample of 872 Americans without prior suspicion of CI.^20^ Our results are comparable regarding our mean age of 66 years in our sample versus 66.8 years in Federman *et al,* as well as the sex distribution with more females in both studies.^20^ Federman *et al* diagnosed CI in 20.8% of participants (mild CI in 10.5% and moderate-to-severe CI in 10.3%),^20^ whereas we identified in our sample 62% mild CI. These differences could be explained by the fact that in our sample, 22% of the participants met difficulties during the test (Table 3).

However, Stimmel *et al* used the English and Spanish version of MoCA among 231 participants in an outpatient primary care setting. The participants had a mean age of 73 years (72% women) and similar results were found to the present study for mild CI with 57% diagnosed (*n* = 133).^21^

Nevertheless, we must keep in mind that the MoCA may sometimes lead to high false-positive rates in ethnoculturally and linguistically diverse settings.^21^

Among our sample, six participants (12%) had hearing problems. The team of the MoCA creator has meanwhile developed and validated the MoCA for people with hearing impairment (MoCA-H), which is a sensitive and reliable means of identifying dementia among adults with acquired hearing impairment.^22^

In another study of Federman *et al*, depression was strongly associated with previously undetected CI among a sample of 855 participants with a mean age of 66.8 years, so again comparable with our sample. We ignore prevalence of depression in our sample, as far as our main objective was to perform the MoCA during routine consultations.^23^

### Implications for practice

Many people are worried about CI, either because of family history or because it is a frightening condition, which is sometimes difficult to discuss with GPs. As a result, most GPs believe that CI is under-screened in general practice.

The suggestion of a screening test to detect mild CI by the GP may help to reassure patients through a dialogue about prevention of a possible risk of social isolation.

The ease of use of the MoCA, combined with the ability to score it, will optimise early screening for CI in general practice.

GPs found the MoCA to be quick and easy to use and patients were satisfied with this cognitive screening consultation. To promote the MoCA use in general practice, it might be worth including it in the restrictive list defined by the NHIF for cotation as a test for early screening for mild CI.

It is important to optimise screening conditions for CI in general practice and Table 4 outlines the points to consider.

**Table 4.** How to optimise screening conditions for cognitive impairment in general practice before the screening

1. Inform the patient clearly and fairly about the nature of the test and possible consequences, such as complementary exams if needed^3^
2. Obtain patient’s consent for the test^3^
3. Ensure that there has been no acute somatic or psychological episode^4^
4. Anticipate that patients may have difficulty concentrating
5. Ensure that there are no visual or hearing impairments that may lead to bias^13^
6. The trustful relationship between the GP and the patient facilitates this screening

Screening for CI is not limited to a single test but an overall assessment, based on a range of clinical, cognitive, functional, and behavioural factors.^13^ Repeating the tests gives confidence in carrying out the procedure, which then quickly becomes part of the GP’s daily practice. However, we need to be aware of the harms of screenings such as leading to overdiagnosis.^24^

If a CI is detected, the GP should carry out a full biopsychosocial assessment of the patient, including close family and friends. The next step is that the GP will provide appropriate support to ensure the patient’s independence for as long as possible through therapeutic training for the patient and caregivers, and the introduction of assistive devices.^5^ As the disease progresses, respite platforms may be used, before, if necessary, early admission to an appropriate institution could be considered.^1^

In conclusion, this study confirms MoCA is quick and easy to use during routine consultations in general practice to screen for early CI for patients aged ≥50 years. Sixty-two per cent of the patients had mild CI, highlighting the importance of early screening to promote early support for patients and their close family and friends.

## Data

Datasets are available from the corresponding author on reasonable request. All data are presented in the article.
